# Double trouble: preseptal cellulitis due to two species with multidrug resistance

**DOI:** 10.1186/1746-160X-9-17

**Published:** 2013-06-26

**Authors:** Muhammad Naeem, Nasir Ali Rahimnajjad, Irfan Majeed, Khalid Mahmood

**Affiliations:** 1 Interns, Dow Medical College, Dow University of Health Sciences, Karachi, Pakistan; 2 Professor and Chief, Department of Internal Medicine, Dow Medical College, Dow University of Health Sciences Karachi, Karachi, Pakistan

## Abstract

A 2 Year old boy presented with painful ballooning of both eyes with the 2 days history of trauma to the head while playing. His vaccination was complete. On examination he was afebrile. The Eyes were ballooned with blackish crust over both lids. On local examination, eye swelling was tense with severe tenderness. The diagnosis of Preseptal cellulitis was made .We did an Emergency drainage and pus was sent for culture that came out to be positive for Pseudomonas aeruginosa and Proteus mirabilis with multiple drug resistance. The coverage was given by Imipenem + cilastatin and child had wonderful recovery.

## Letter to the Editor

**Figure 1:**
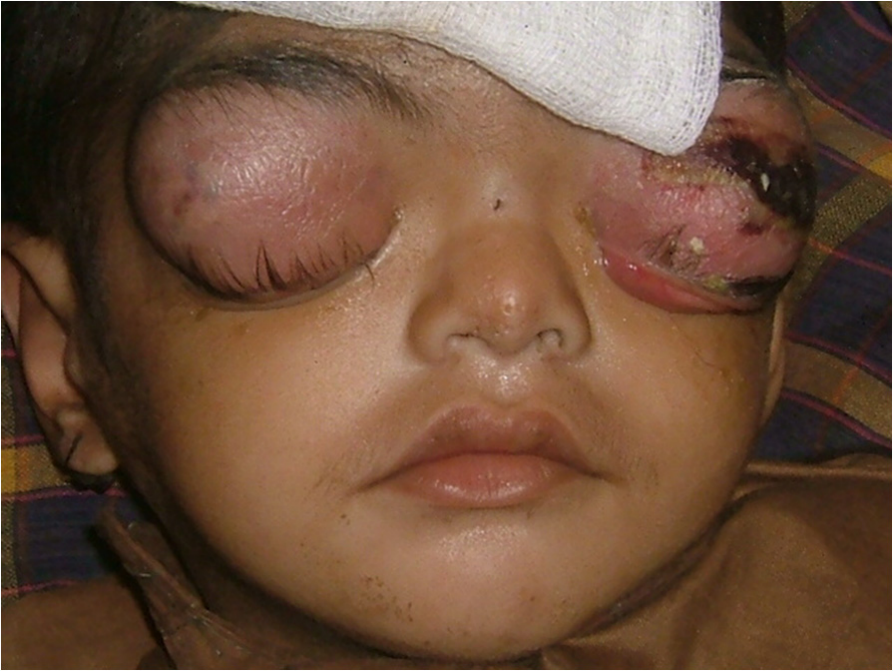
Photograph showing the patient’s eyes with preseptal cellulitis.

A 2 Year old boy presented with painful ballooning of both eyes with the 2 days history of trauma to the head while playing. His complete history reveals unremarkable antenatal, natal and postnatal history. He was afebrile on presentation. His vaccination was complete according to EPI schedule. The past history was significant for sinusitis for which he got the treatment (undocumented). On examination the patient was afebrile with dry cough and vitally stable. The Eyes were ballooned with blackish crust over both lids (Figure 1). On local examination, eye swelling was tense with severe tenderness. His blood work up was normal except raised leukocyte count with left shift (might be due to infection). The diagnosis of pre-septal cellulitis was made after excluding other possible causes clinically and radiologically and antibiotic was empirically started (amoxicillin + clavulinic acid) after both eyes were drained and pus was sent for culture and sensitivity, the culture report came out to be positive for Pseudomonas aeruginosa and Proteus mirabilis with multiple drug resistance (Table 1). As indicated in the antibiogram, the coverage was given by Imipenem + cilastatin (15/15 mg/kg/dose every 6 hours) because it was the only drug to which both bacteria were sensitive. The child had wonderful recovery after the treatment. Written informed consent was obtained from the patient’s parents for the publication of this report and any accompanying images.

**Table 1:** Resistance antibiogram

**S. no**	**Antibiotic**	**Sensitivity patterns**	
		**Pseudomonas aeruginosa**	**Proteus mirabilis**
**1-**	Ampicillin	R	R
**2-**	Co-amoxiclav	R	R
**3-**	Imipenem & Cilastatin	S	S
**4-**	Cefixime	R	R
**5-**	Ceftriaxone	R	R
**6-**	Ceftazidime	S	R
**7-**	Cefuroxime	R	S
**8-**	Norfloxacin	R	R
**9-**	Ciprofloxacin	S	R
**10-**	Levofloxacin	S	R
**11-**	Gentamicin	R	R
**12-**	Tobramycin	S	R

R = Resistant.

S = sensitive.

Preseptal cellulitis is characterized by hyperemic skin and eyelid distension without the obvious signs of orbital congestion [1]. Preseptal cellulitis can occur as a result of exogenous as well as endogenous extension [2]. In this particular case the rare combine infection by two bacteria with multi drug resistance made this case interesting and difficult to manage. This infection can progress to involve the post-septal space and result in orbital cellulitis with life threatening complications. Majority of the cases are caused by Streptococci or Staphylococci [3]. While making diagnosis, this condition should be differentiated from other causes of Eye swelling ranging from simple inflammation to life threatening Retinoblastoma [4]. Once this diagnosis is suspected it should be related with lab investigations like complete blood count, blood culture and culture of aspirate so that therapy should be targeted [5]. Computed Tomography is recommended investigation to delineate the extension of the cellulitis [6]; however, is not necessary. Nasal decongestants are recommended if sinusitis is suspected and antibiotics should be continued from three to ten days depending on severity of the infection.

Vision is the most wonderful sense gifted by nature and restoration of vision after such sinister infection was the source of joy both for family and doctors.

## Competing interests

The authors state that they have no competing interest.

## Authors’ contribution

MN and NAR: literature search and conception. IM and NAR: Initial draft preparation. KM and MN: Finalization of the draft. Picture and Informed Consent taken: MN. Informed Written consent was taken for the publication of the picture. All authors read and approved the final manuscript.
